# Genome editing in the edible fungus *Poria cocos* using CRISPR-Cas9 system integrating genome-wide off-target prediction and detection

**DOI:** 10.3389/fmicb.2022.966231

**Published:** 2022-08-22

**Authors:** Zhenni Xie, Can Zhong, Xiaoliu Liu, Ziling Wang, Rongrong Zhou, Jing Xie, Shuihan Zhang, Jian Jin

**Affiliations:** ^1^Graduate School, Hunan University of Chinese Medicine, Changsha, China; ^2^Institute of Chinese Materia Medica, Hunan Academy of Chinese Medicine, Changsha, China

**Keywords:** medicinal fungus, *Poria cocos*, CRISPR/Cas9, genome editing, endogenous promoter, off-target

## Abstract

*Poria cocos* is an important edible and medicinal fungus with a long history. However, the lack of adequate genetic tools has hindered molecular genetic research and the genetic modification of this species. In this study, the endogenous U6 promoters were identified by mining data from the *P. cocos* genome, and the promoter sequence was used to construct a sgRNA expression vector pFC332-PcU6. Then, the protoplast isolation protocol was developed, and the sgRNA-Cas9 vector was successfully transformed into the cells of *P. cocos via* PEG/CaCl2-mediated transformation approach. Off-target sites were genome-widely predicted and detected. As a result, the target marker gene *ura3* was successfully disrupted by the CRISPR-Cas9 system. This is the first report of genome editing in *P. cocos* using CRISPR-Cas9 system integrating genome-wide off-target prediction and detection. These data will open up new avenues for the investigation of genetic breeding and commercial production of edible and medicinal fungus.

## Introduction

*Poria cocos* (=*Wolfiporia cocos* or *Wolfiporia extensa*), named Fuling in Chinese, is an edible and medicinal fungus famous along with the valuable Ganoderma, Ginseng, Dendrobii, and Cordycep*s* (Wang et al., 2013; Jin et al., 2020a). Its sclerotia, consisting of compact masses of hardened mycelia, are regarded as an important source of medicine in Asia and also known as “Indian bread” in North America (Lee et al., 2018). This fungus demonstrated various pharmacological properties, such as antitumor, immunomodulatory, anti-inflammatory (Kim et al., 2019). It is widely used as raw material in Chinese classical prescriptions, patent medicine, and functional foods (Jin et al., 2020b). With great demands in China and overseas markets, the production of *P. cocos* is about 35,000 ton/year and 4,000 ton/year exporting from China to Japan, Korea, Vietnam, Malaysia, and dozens of countries (Jin et al., 2022).

With regard to the important edible resources, advanced biotechnology and genetic engineering usually play a critical role in the rapid creating and developing of excellent germplasm. *P. cocos* as a filamentous fungus has a genetic background that is more complex than those of other multicellular eukaryotic organism and bacteria, resulting in their genetic manipulation is often difficult. This has led to relatively slow progress in molecular biology and genetic research of filamentous fungi (Song et al., 2019). Most of the studies of *P. cocos* focused on its phytochemistry and pharmacological activities mainly the isolation process, structural features, and bioactivities of triterpenes and polysaccharides (Jia et al., 2016; Nie et al., 2020). Although some investigations of cloning the key enzyme genes such as squalene synthase gene (Wang et al., 2014b), farnesyl diphosphate synthase (Wang et al., 2014a), and cytochrome P450 (He et al., 2016) being carried out in *P. cocos*, much work to be done to find the effective genetic toolbox for this valuable edible resource.

Clustered regularly interspaced short palindromic repeats (CRISPR) system with its associated nuclease Cas9 was a newly innovative and efficient genome-editing tool with gene knockout, insertion, and replacement abilities (Nidhi et al., 2021). This vital site-specific gene editing tool was developed in 2013 and awarded the Nobel Prize in Chemistry in 2020 (Li et al., 2021a). It only requires a short guide RNA (sgRNA) sequence to recognize the target site according to nucleotides base, the endonuclease activity of Cas9 can lead to gene modification by cleaving the target DNA, forming DNA double-strand breaks, and stimulating DNA repair mechanisms *in vivo*, resulting in gene mutation (Xuan et al., 2017). CRISPR-Cas9 system has been rapidly developed and successfully applied to alter metabolic pathways, improve Chinese Materia Medica quality and drug development *via* gene mutation, such as in *Cordyceps militaris* (Chen et al., 2018), *Dendrobium officinale* (Ling et al., 2017), *Shiraia bambusicola* (Deng et al., 2017), *Ganoderma lucidum* (Wang et al., 2020), *Rehmannia glutinosa* (Li et al., 2021b), and *Salvia miltiorrhiza* (Zhou et al., 2018).

Although the CRISPR-Cas9 system could be an adequate genetic toolbox applied to *P. cocos* genome editing, there are still some obstacles to be overcome. In the sgRNA-Cas9 binary vector system, an efficient promoter to facilitate sgRNA transcription *in vivo* is a necessity (Nidhi et al., 2021). The sgRNA in the CRISPR/Cas9 system does not require a cap structure or a polyA tail. In most CRISPR-Cas9 systems, sgRNA is driven by RNA polymerase type III U6 promoters that produce small, non-polyadenylated, nuclear RNAs. The endogenous U6 promoters have been characterized and applied to transcribe gRNA for CRISPR-Cas9 genetic manipulation in some fungi, such as in the traditional medicinal mushroom *G. lucidum* (Qin et al., 2017), the rice blast fungus *Pyricularia oryzae* (Arazoe et al., 2015), and the fungal maize pathogen *Ustilago maydis* (Schuster et al., 2016). However, the U6 promoter has not been identified and validated in *P. cocos*. Therefore, firstly, it is important to find reliable promoters to drive sgRNA transcription *in vivo* and to test their utility for efficient genome editing of *P. cocos* (Wang and Rollins, 2021).

Another challenge is off-target effect that Cas9 binds to unintended genomic sites for cleavage (Singh et al., 2016). Identifying the potential off-target sites is important for users to effectively use the system to edit genomes (Pavese et al., 2022). The available genome sequence of host species could help select target sites with low off-target efficiency and increase the accuracy of genome editing (Denes et al., 2021). Most of the current off-targets prediction tools are focused on the model organism, for instance, the *Arabidopsis thaliana*, with well-characterized genome information. Although the genome of *P. cocos* has been published in recent years (Floudas et al., 2012; Luo et al., 2020; Cao et al., 2021), using an off-line tool to predict the off-target sites in *P. cocos*, a non-model filamentous fungus, is not an obvious easy-to-solve task. To the best of our knowledge, so far, there is no genome editing report using CRISPR system in *P. cocos*.

In this study, the application of a CRISPR system in *P. cocos* is reported. We mined data from the *P. cocos* genome to identify the endogenous U6 promoters and used the promoter sequence to construct a sgRNA expression vector. Then, the sgRNA-Cas9 vector was successfully transformed into the protoplast of *P. cocos via* PEG/CaCl2-mediated transformation approach. In addition, genome-wide off-target sites were predicted and detected. As a result, the target marker gene of orotidine 5′-phosphate decarboxylase (*ura3*) was successfully disrupted by the CRISPR-Cas9 system. These data will open up new avenues for the investigation of genetic breeding and commercial production of the edible and medicinal *P. cocos*.

## Materials and methods

### Strains and cultural conditions

Sclerotia of *P. cocos* Xiangjing 28 were collected from Jingzhou County, Hunan Province, China (N 26°43′36.38″, E 109°41′15.34″). The fungus was isolated from the inner part of sclerotia and cultivated on a modified PDA (MPDA) medium that contained peptone (5.0 g/L), glucose (15.0 g/L), KH_2_PO_4_ (1.0 g/L), MgSO_4_⋅7H_2_O (0.5 g/L), yeast extract powder (2.0 g/L), and agar (15.0 g/L). *E. coli* strain DH5a or Stbl 3 (Tsingke, Beijing, China) was used for vector construction, incubated in the LB medium at 37°C, using kanamycin as a selection reagent.

### Endogenous sgRNA promoter identification

The U6 snRNA gene sequence was acquired from the genome of *P. cocos* (data not shown) by INFERNAL software against the Rfam database (Nawrocki et al., 2009). The U6 snRNA gene sequences were aligned by Mega 6 using the U6 snRNA gene of *Aspergillus nidulans* (GenBank accession number: MH032752.1). A 300-nt promoter region (Long et al., 2018) upstream the U6 snRNA gene ORF was obtained by Tbtools (Chen et al., 2020) according to its genome locus. The upstream sequences of predicted U6 promoters from *P. cocos* and *A. nidulans* AnU6 promoter, *A. thaliana* AtU6 promotor (GenBank accession number: MN728556.1) were analyzed by Plant CARE^1^ (Lescot et al., 2002).

### Construction of sgRNA-Cas9 plasmids

A Cas9 constitutive expression vector containing the resistance gene marker of hygromycin, i.e., pFC332 (Nødvig et al., 2015), obtained from Micro-Helix Co., Ltd., was used for the construction of sgRNA-Cas9 cassettes. For the construction, the vector containing endogenous U6 promoter of *P. cocos*, the 300-nt PcU6-1 promotor sequence, the sgRNA site including 76-nt scaffold sequence, and the terminator of 8 Ts were synthesized in Tsingke Biotechnology Co., Ltd., and constructed into pFC332 by *Bgl*II and *Pac*I. To evaluate the efficiency of the CRISPR-Cas9 system in *P. cocos*, the *ura3* gene was chosen as the editing target. The sgRNAs were designed by using the online website^2^ and two target sites (called Ura3-sgRNA1 and Ura3-sgRNA1) were chosen using CRISPick (GPP sgRNA Designer). The sgRNA cassette primers were synthesized by Sangon Biotech in Tsingke Biotechnology Co., Ltd. The target sgRNA sequence of Ura3-sgRNA1 and Ura3-sgRNA2 was constructed into the pFC332-PcU6 vector by Golden gate method using *Aar*I and T4 DNA ligase (NEB) *in vitro* (Xing et al., 2014), namely pFC332-PcU6-Ura3-sgRNA1 and pFC332-PcU6-Ura3-sgRNA2, respectively.

### Protoplast preparation and microscopic observation

Mycelia of *P. cocos* cultivated for 7 days were harvested and washed twice with sterile water, the mycelia were digested by 0.6 M mannitol with 1.0, 1.5, and 2.0% lywallzyme (Guangdong Culture Collection Center, Guangzhou, China) at 30°C, with gentle shaking (85 rpm) for 1, 2, and 3 h, to optimize the protocol. Protoplasts were then filtered and washed in 0.6 M mannitol solution twice. After subsequent washing in MTC buffer (0.6 M mannitol, 50 mM CaCl_2_, 10 mM Tris-Cl pH 7.5), the protoplasts were resuspended in MTC buffer at a density of 10^6^ protoplasts in each milliliter. During the protoplast preparation process, it was observed step by step with the microscope (MSD 105, Guangdong, China) with 400 times magnification to confirm the release of protoplast from mycelia.

### PEG-mediated protoplast transformation

The PEG-mediated protoplast transformation was modified as previously described (Sun et al., 2015). Each 100 μL of protoplasts was mixed with constructed sgRNA-Cas9 plasmid and placed on ice for 30 min. A volume of 1000 μL of PEG buffer (PEG 4000 45%, 50 mM CaCl_2_, 10 mM Tris-Cl, pH 7.5) was slowly added. The whole mixture was incubated for 15 min on ice, and then mixed with 2000 μL of MTC buffer and transferred to a plate. Then, MPDA medium with 0.6 M mannitol and 0.05 mM uracil was added to the plate and incubated at 28°C for 3 days of regeneration. After that, MPDA medium with hygromycin and uracil was used to directly cover the surface. When the mycelia grown out of the medium, the mycelia transferred to cultivation on other plate embedded in MPDA medium with hygromycin, 5-fluoroorotic acid (5-FOA) and uracil, since the disruption mutations in Ura3 could cause auxotrophy for uridine (Chen et al., 2018).

### Identification of resistance marker

To identify the resistance marker for detecting the transformants, the mycelia of *P. cocos* wild type were inoculated onto plate with different concentrations of hygromycin (0, 0.025, 0.0625, 0.125, 0.175 mg/mL) or 5-FOA (0, 0.020, 0.060, 0.100, 0.200 mg/mL) in medium for cultivation 30 days, to observe the inhibition effect of the growth.

### Genomic DNA extraction and mutation identification

Until the mycelia had grown out of the medium with hygromycin or 5-FOA, individuals were picked and tested by PCR verification. The genomic DNA of *P. cocos* mycelia was extracted by Cetyltrimethyl Ammonium Bromide (CTAB) method (Brandfass and Karlovsky, 2008). To identify whether the constructed plasmid had successfully transformed into the mycelia, we firstly designed specific primers to test the sgRNA regions (sgRNA-F and sgRNA-R) and *cas9* (cas9-1-F and cas9-1-R) of plasmid by PCR amplification from the mycelia grown on the plate with hygromycin. For the mutation identification of the *P. cocos* mycelia grown on the plate with 5-FOA, the designed target sites in *ura3* gene were amplified using the primer pair (ura3-F and ura3-R), of which the binding positions are at about 400 bp upstream or downstream of the target sites (Table 1). Then, agarose gel electrophoresis was performed to verify the target bands. PCR products were validated through sequencing by Tsingke Biotechnology Co., Ltd., and wild *P. cocos* strain was chosen as control. Sequence chromatograms of heterozygous and biallelic mutations were decoded using Degenerate Sequence Decoding method (Ma et al., 2015). The efficiency of genome editing was evaluated as the number of positive mutant clones divided by the total number of clones (Arazoe et al., 2015).

**TABLE 1:** The primers for PCR and qRT-PCR analysis.

No.	Name of the primer	Sequence
1	sgRNA-F	ATGCCTCTTGGGCTGGTAAC
2	sgRNA-R	GCCTCTACGGTGGATTCTCG
3	cas9-1-F	GAAGATGAAGAACTACTGGC
4	cas9-1-R	CCCTTCTCAACCTTCGC
5	ura3-F	GAAGACTTTGAGCCCTCGCT
6	ura3-R	GATGAACCCGACGACGAAGT
7	CYP-F	CATGGCTTCGGCTACAAGG
8	CYP-R	TTGGTGTGCTTGAGCTTGAA
9	qRT-PCR-sgRNA1-F	GGCGTCTACAAAATCGCCAGC
10	qRT-PCR-sgRNA2-F	GGTCCTGGCACAGGGTGCGCGT
11	qRT-PCR-sgRNA-R	CAAGTTGATAACGGACTAGCCT
12	qRT-PCR-cas9-F	GGTTGGGCTGTGATTACGGA
13	qRT-PCR-cas9-R	CAGATGCGATTCTTGCGTCG

### Expression analysis of sgRNA and Cas9 by qRT-PCR

The RNAprep Pure Plant Plus Kit (TianGen Biotech, Beijing, China) was used to extract total RNA from the mycelium, and DNA contamination was eliminated by DNase treatment. The quality and concentration of the obtained RNA were measured by ScanDrop 100 (Analytik jena, Germany), and then the RNA was reverse transcribed to complementary DNA by a Goldenstar RT6 cDNA Synthesis Kit (Tsingke Biotech, Beijing, China). The premiers for quantitative real-time reverse transcription PCR (qRT-PCR) are listed in Table 1, and qRT-PCR was performed following the instructions of the T5 Fast qPCR Mix (Tsingke Biotech) on a CFX Connext Optics Module Real Time System (Bio-Rad Inc., Singapore). The efficiency-corrected comparative threshold cycle (CT) method was applied, and relative expression was calculated by Delta CT method of normalizing the genes of sgRNA1, sgRNA2, and Cas9 according to the abundance of the reference gene cyclophilin (CYP) (Zhang et al., 2016).

### Genome-wide off-target prediction and detection

To determine whether off-target events had occurred in *P. cocos*, a pair of specific primers were designed using the off-target prediction by Tbtools with the genome data to amplify potential off-target DNA fragments. The algorithm program was set as blasting the 15-bp sequence, the 12-bp from the seed sequence of sgRNA, and 3-bp Nitrogenous-base (Adenine or Guanine or Cytosine or Thymine) Guanine Guanine (NGG) Protospacer Adjacent Motif (PAM) motifs to the whole genome with no or one mismatches of Ura3-sgRNA1 and Ura3-sgRNA2, which are reported as the usual off-target sites (Le et al., 2013). Then, the primer pairs were designed as a list in Table 2 by Primer-BLAST to amplify the potential off-target sites. The off-target sites were verified by comparing the sequencing data of PCR products to that of the wild *P. cocos* strain.

**TABLE 2:** The primers for the detection of potential off-target site by PCR amplification.

sgRNA	No.	Name of the primer	Sequence
Ura3-sgRNA1	1	OTS1-1 F	TCTGGCATCTCCAACCCTCT
	2	OTS1-1 R	CCCACTTACAATGGCACGCT
	3	OTS1-2 F	GCCGTGACAACTACTCGCTA
	4	OTS1-2 R	CGGTGAAATGAATCTGCGGC
	5	OTS1-3 F	GCGGAGATGTCAACAAAGCC
	6	OTS1-3 R	CAAGAAGCCACTCGTTCAGC
	7	OTS1-4 F	AGCGGTATGTTGTGGTGTCC
	8	OTS1-4 R	CGGCGTGAATTTGCTCACTG
	9	OTS1-5 F	CCACACTCTGCCAACCATGA
	10	OTS1-5 R	TGGTCGTGTTATGCGAGACC
Ura3-sgRNA2	1	OTS2-1 F	TTCGGGCAGACTTGGATCAC
	2	OTS2-1 R	TGTTCAGGGAAACCGTAGCC
	3	OTS2-2 F	TTACCTGTGGCGCGAGTATG
	4	OTS2-2 R	GTGGGTACCGGGATAACGAG
	5	OTS2-3 F	ATAACGAGGTCAAGTCCGGC
	6	OTS2-3 R	TTACCTGTGGCGCGAGTATG
	7	OTS2-4 F	GAAACCGTGGCCGATCATTG
	8	OTS2-4 R	TTCGGGCAGACTTGGATCAC
	9	OTS2-5 F	CGCACGTCAGATGGCAAAAA
	10	OTS2-5 R	AGTGCAGTGAAGTACCCAGC

### Statistical analysis

All statistical analysis was performed in triplicate, and the values were expressed as the mean ± standard deviation. Differences between groups were compared by one-way ANOVA, followed by LSD. These analyses were performed with the statistical tool of the Origin software. Differences were considered to be significant in all statistical tests with a *P* < 0.05.

## Results

### Analysis of sgRNA promoter from genome of *Poria cocos*

**FIGURE 1:**
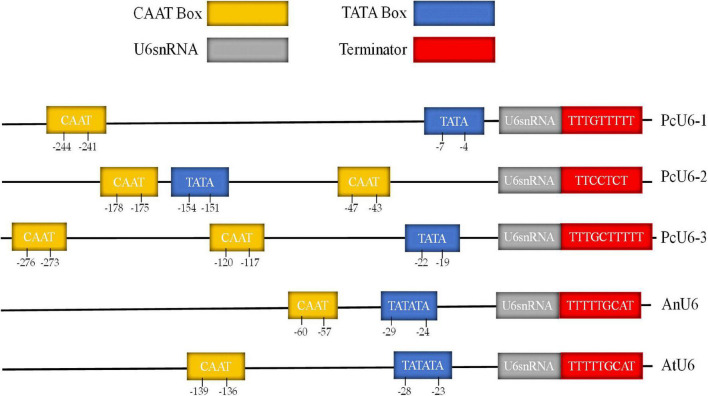
The comparative analysis of promoter and terminator of U6 snRNA genes of the species *Poria cocos, Aspergillus nidulans*, and *Arabidopsis thaliana*.

The sgRNA transcribed by U6 promoter could enhance the gene editing efficiency of CRIPSR-Cas, because the pol III promoters adopt a thymidine (T)-stretch as a termination signal, yielding small RNA transcripts with different lengths of U-tails having a negative effect on the functionality of CRISPR system (Wang et al., 2020). Three putative U6 snRNA genes were identified with termination sequences consisting of several Ts from the *Poria cocos* genome database by a blast search (Lambeth et al., 2005). The three genes were randomly designated PcU6-1, PcU6-2, and PcU6-3 snRNA (Figure 1). The nucleotide sequences of all three U6 snRNA genes are relatively conserved and showed high homology comparing to the U6 snRNA sequence of *Aspergillus nidulans*. However, the upstream promoter region diverged significantly (Supplementary Figure 1). Although the typical TATA-box and CAAT-box were found in all these promoter sequences (Hu et al., 2011), other elements usually presented in animal U6 promoter such as the proximal sequence elements (PSE) consensus sequences with conserved regional ACCAC in zebrafish (Boonanuntanasarn et al., 2009) or GTGCGAATGAGTTAT TGAATG in *Schistosoma japonicum* (Li et al., 2017), and octamer motif (ATTTGCAT) were not obvious observed.

### Construction of endogenous PcU6 promoter vector

**FIGURE 2:**
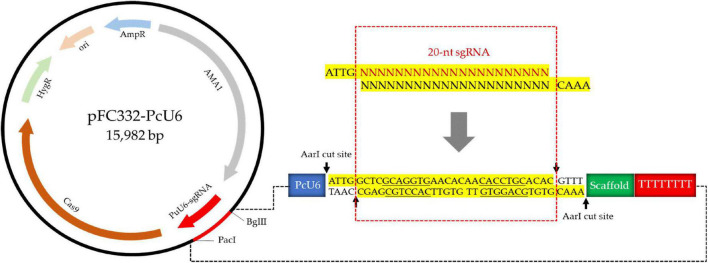
The schematic of construction of pFC332-PcU6 plasmid including the endogenous U6 promoter, sgRNA site containing *Aar*I restriction sites, the sgRNA scaffold sequence, and the terminator.

For the construction of vector containing endogenous U6 promoter of *P. cocos*, the 300-nt promotor sequence of PcU6 and the sgRNA site containing *Aar*I restriction sites, the sgRNA scaffold sequence, and the terminator of 8 Ts were synthesized and formed the pFC332-PcU6 vector (Figure 2). Every element is essential in this vector except the extra spacer between two *Aar*I restriction sites in sgRNA expression cassette, which are designed for the integration of 20-nt sgRNA fragments. The constructed plasmid is 15,982-bp in length and stored in *Escherichia coli* Stbl 3, noting that it was unstable to store this plasmid in the *E. coli* strain DH5a. Then, this plasmid was used for future construction of the sgRNA-Cas vectors.

### Obtaining the protoplast and identification of resistance marker

**FIGURE 3:**
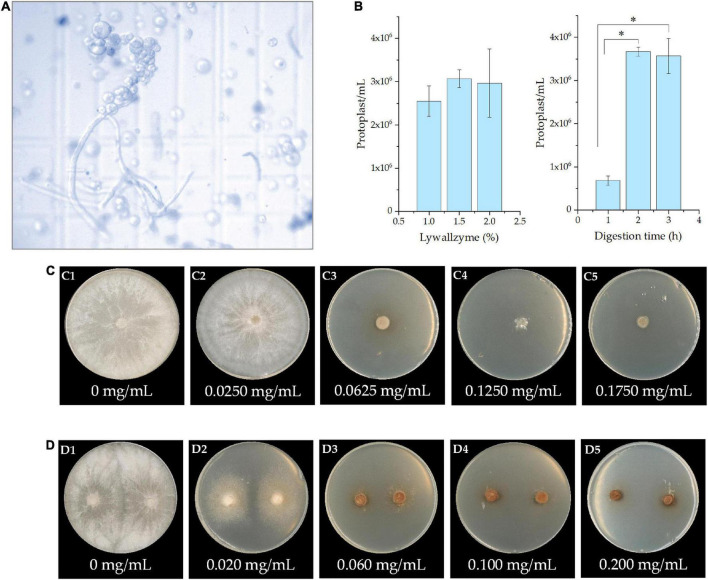
The protoplast and identification of resistance marker of *Poria cocos*. **(A)** The microscopic observation of protoplasts obtained from the mycelia by lywallzyme digestion; **(B)** the optimization of concentration of lywallzyme and digestion time for protoplasts preparation; **(C)** the resistance marker selection of hygromycin; and **(D)** the resistance marker selection of 5-FOA at different concentration; *P < 0.05.

The mycelia of *P. cocos* were well digested by the lywallzyme, and protoplasts were released into the buffer solution (Figure 3A). In order to develop the protoplast isolation protocol, the concentration of lywallzyme and the digestion time was optimized. As shown in Figure 3B, there is no significant difference between the concentration of lywallzyme at 1.0 and 2.0%. Whereas the protoplasts density at the digestion time of 2 or 3 h was significantly higher than that at 1 h (*P* < 0.05). No obvious difference was observed between digestion of 2 and 3 h. Therefore, protoplasts at a density of 10^6^/mL were obtained for further investigation by digesting mycelia for 2.0 h with 1.5% lywallzyme.

The effect of the minimum inhibitory concentration of hygromycin or 5-FOA on *P. cocos* was determined by cultivating the strain on MPDA plates with different drug concentrations for a month. As shown in Figure 3C, hygromycin has a toxic effect on *P. cocos* strain Xiangjing 28 at a testing dose up to 0.0625 mg/mL. The medium with 5-FOA showed obvious lethality in the mycelia of *P. cocos*. The growth of wild-type mycelia was inhibited at a 5-FOA concentration of 0.0600 mg/mL (Figure 3D). To prevent false positive result, the medium with relatively high concentrations, i.e., the hygromycin at concentration 0.125 mg/mL and 5-FOA at concentration 0.200 mg/mL, was chosen for further study.

### Transformation of sgRNA-Cas9 plasmid into *Poria cocos*

**FIGURE 4:**
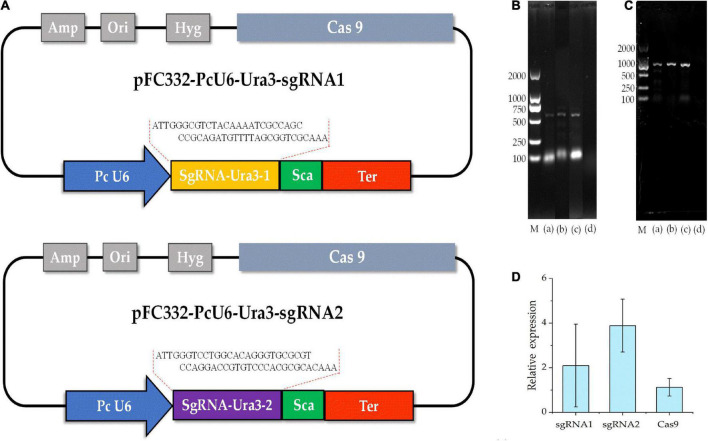
The diagram of CRISPR-Cas9 system with different sgRNA target sites and detection of sgRNA and Cas9 of the transformants. **(A)** The schematic of the vector pFC332-PcU6-Ura3-sgRNA1 and pFC332-PcU6-Ura3-sgRNA2; **(B)** agarose gel electrophoresis of PCR products of sgRNA; **(C)** agarose gel electrophoresis of PCR products of Cas9; M, DNA ladder 2000 Marker; (a) plasmid; (b) ura3-sgRNA1 transformants; (c) ura3-sgRNA2 transformants; (d) wild type; and **(D)** the relative expression of the genes of sgRNA1, sgRNA2, and Cas9.

To detect the genome editing efficiency of the CRISPRCas9 system, two target sites were chosen in the *ura3* marker gene locus (Figure 4A). The plasmid pFC332-PcU6-Ura3-sgRNA1 and pFC332-PcU6-Ura3-sgRNA2 were transformed into the *P. cocos* by the PEG-mediated protoplast transformation. When the mycelia grown on the medium with hygromycin, the sgRNA regions and partial sequence of Cas9 gene were detected in both *P. cocos* transformants of sgRNA1 and sgRNA2 by PCR amplification (Figures 4B,C), indicating these two plasmids were successfully transformed into *P. cocos* cells. As shown in Figure 4D, the genes of Ura3-sgRNA1, Ura3-sgRNA2, and Cas9 were found expressed in *P. cocos* comparing to the reference gene CYP by a qRT-PCR approach.

### Establishment of CRISPR-Cas9 gene disruption system by different sgRNA target sites

**FIGURE 5:**
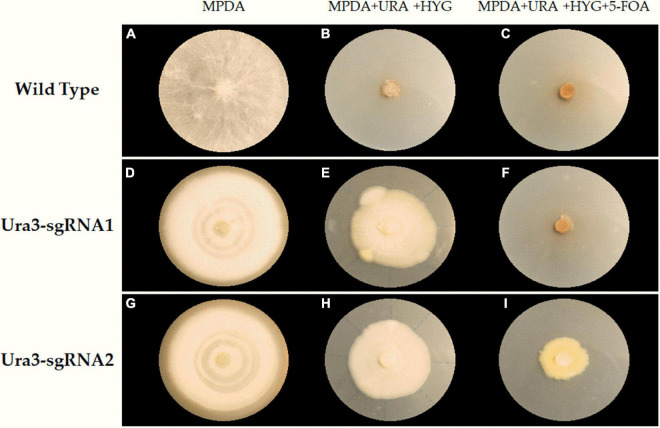
The selection of transformants and mutants on a different medium plate. **(A)** Wilde type grown on MPDA; **(B)** wilde type grown on MPDA + URA + HYG; **(C)** wilde type grown on MPDA + URA + HYG + 5-FOA; **(D)** Ura3-sgRNA1 grown on MPDA; **(E)** Ura3-sgRNA1 grown on MPDA + URA + HYG; **(F)** Ura3-sgRNA1 grown on MPDA + URA + HYG + 5-FOA; **(G)** Ura3-sgRNA2 grown on MPDA; **(H)** Ura3-sgRNA2 grown on MPDA + URA + HYG; **(I)** Ura3-sgRNA2 grown on MPDA + URA + HYG + 5-FOA; Wild type, the wild type of *Poria cocos* strain; Ura3-sgRNA1, the transformant of pFC332-PcU6-Ura3-sgRNA1; Ura3-sgRNA2, the transformant of pFC332-PcU6-Ura3-sgRNA2; MPDA, the modified PDA medium; MPDA + URA + HYG, MPDA medium with hygromycin and uracil. MPDA + URA + HYG + 5-FOA, MPDA medium with hygromycin, 5-FOA, and uracil.

**FIGURE 6:**
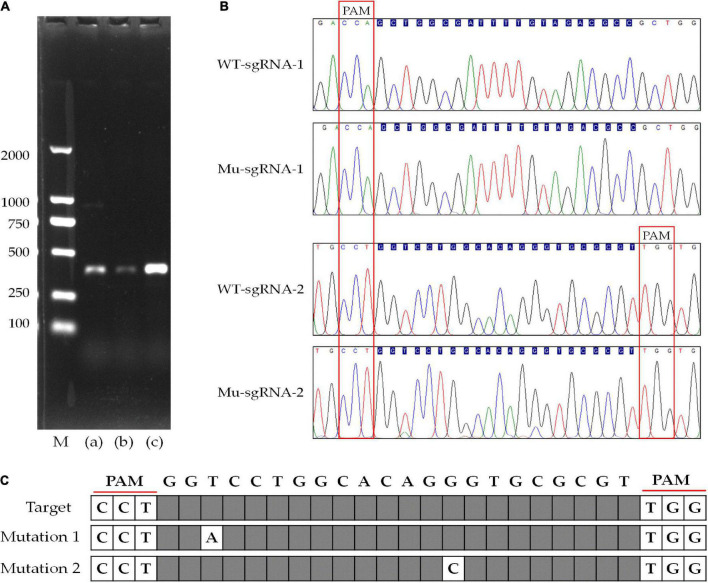
Disruption of the gene targeting to *ura3* by CRISPR-Cas9 system with different sgRNA target sites. **(A)** Agarose gel electrophoresis of PCR products of *ura3* in wild-type and mutant strain; **(B)** sequencing chromatogram of the Ura3-sgRNA1 and Ura3-sgRNA2 target region; **(C)** the display of the mutated site at Ura3-sgRNA2 target region; M, DNA ladder 2000 Marker; (a) sgRNA-1 target site of *ura3*; (b) sgRNA-2 target site of *ura3*; and (c) wild type of *ura3* including sgRNA-1 and gRNA-2.

The compound 5-FOA is a commonly used negative selector marker for fungi (Qin et al., 2017), because the enzyme Ura3 will convert 5-FOA into a toxic compound and lead to cell death, and the disruption phenotype of which was 5-FOA resistant and could be easily observed. As shown in Figure 5, the transformants were transferred to further cultivation on a medium with 5-FOA to select the mutants. Whereas we only obtained transformants that were grown out of the 5-FOA medium in the pFC332-PcU6-Ura3-sgRNA2 transformants, even though with a slow growth rate. The target sites in wild-type and mutant strains were obtained by PCR amplification, respectively. Sequencing results have shown that Ura3-sgRNA2 target sites were mutated around the CRISPR-Cas9 editing region, implying the *ura3* gene was successfully disrupted by the CRISPR-Cas9 system. Meanwhile, the double peaks in the sequencing chromatogram were resulted from heterozygous character of *P. cocos* with only one copy nuclei edited. As shown in Figure 6, the mutation was a base transformation that changing the C to G or A to T of Ura3-sgRNA2 target, resulting in the missense mutation of changing alanine to proline at position 125 in Ura3 protein. By the way, the PCR amplification of the Ura3-sgRNA1 site of the transformants from the medium with hygromycin was not found with mutation comparing the target region sequence to the wild *P. cocos* strain. By calculating the positive mutant clones divided by the total number of clones, the targeting editing efficiency of *ura3* was evaluated as 1.92%.

### Genome-wide off-target prediction and detection

**FIGURE 7:**
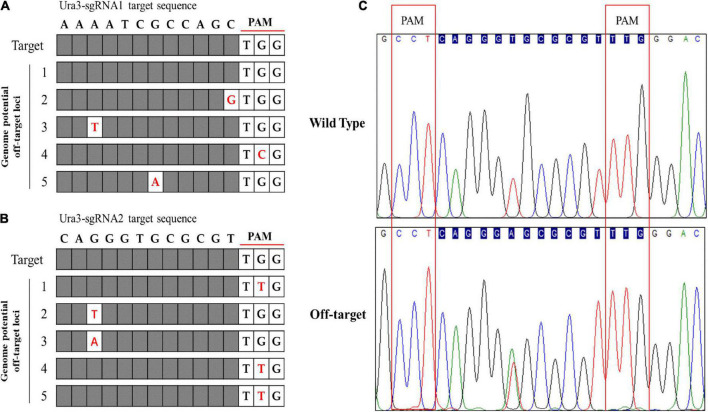
Genome-wide potential off-target prediction with no or one mismatch and sequencing detection; **(A)** the potential off-target site of Ura3-sgRNA1; **(B)** the potential off-target site of Ura3-sgRNA2; and **(C)** sequencing chromatogram of the detected off-target region comparing the mutant strain to the wild type strain.

The off-target effects are commonly due to PAM and sgRNA mismatches. Currently, various bioinformatics tools have been developed to help predict and reduce off-target modifications (Zhang et al., 2022). In this study, the potential off-target sites of CRISPR/Cas9 were predicted through 12 nucleotide sequences of sgRNA and PAM sites by aligning the sgRNA sequences with reference genome sequences using Tbtools software to search mismatch. Five potential off-target sites were identified with one mismatch to Ura3-sgRNA1 and Ura3-sgRNA2, respectively (Figures 7A,B). Using the genomic DNA of *P. cocos* transformants as a template, the potential off-target sites were amplified by primer pairs. Sequencing results have shown that the five potential off-target sites of Ura3-sgRNA1 were not mutated around the CRISPR-Cas9 editing region. Meanwhile, it is worth to mention that there is a potential off-target site of Ura3-sgRNA2 indeed mutated by PCR detection, by comparing the sequencing chromatograph of the mutant strain to that of the wild type (Figure 7C). Interesting, not only the on-target site but also the off-target site successfully mutated with the NGG PAM at both sides of the sgRNA, indicating to design the sgRNAs with PAM motifs at the DNA sense and antisense strands would be meaningful for the improvement of the genome editing efficiency.

## Discussion

Some researchers have constructed different binary vectors by combining Cas9 with gRNA and induced target gene modification. RNA polymerase III promoters have been widely used to express short hairpin-RNA (shRNA), microRNA (miRNA), and sgRNA in gene functional analysis in a variety of organisms (Nidhi et al., 2021). However, no endogenous RNA polymerase III promoters have been identified in *P. cocos*, which hindered its gene functional analysis. Identification of functional promoters in *P. cocos* is therefore in urgent need (Li et al., 2017). We have mined data from the *P. cocos* genome and three endogenous U6 promoter were identified. Although these gene sequences of RNA polymerase III (pol III) might be conserved in eukaryotes (Wang et al., 2020), much work remains to be done to characterize the functional box. TATA box was reported critical for the transcription of pol III in eukaryotes to effectively promote shRNA expression (Gao et al., 2018). While the PSE consensus and octamer motif could be different, at least these conserved sequences were not obvious observation in the U6 promotor of *P*. *cocos*. By the way, this octamer motif was also not present in U6 snRNA gene in other organisms taking *Drosophila* as an example (Boonanuntanasarn et al., 2009).

To detect the efficiency of the CRISPRCas9 system, two target sites were chosen in the *ura3* gene locus because Ura3 will convert 5-FOA into a toxic compound and lead to cell death. The efficiency of genome editing could be varied for different species. For example, an gene editing efficiency of 36.1–80.5% was achieved using endogenous U6 promoter in the model filamentous fungus *Pyricularia oryzae* (Song et al., 2019), whereas successful disruption of functional genes using a codon optimized Cas9 vector in *G. lucidum* demonstrated an efficiency of 22.2% (Wang et al., 2020). In this study, the gene editing efficiency seems also to be site dependent. Of the two sgRNA target sites, only the Ura3-sgRNA2 target site was the successfully generated mutation. This is also found in other organisms, for instance, target sites have different mutation rates and some targets totally fail to be mutated in *D. officinale* (Ling et al., 2017). The low efficiency in *P. cocos* could be resulted from the Cas9 protein. Cas9 system was originally discovered in bacteria or Archaea. Therefore, when the CRISPR/Cas9 system is used in fungi, the Cas9 protein is usually fungal codon optimized, and a nuclear localization signal is added at both ends of the Cas9 gene (Song et al., 2019). The Cas9 cassette used in this study is in the vector pFC332, which is from *Streptococcus pyogenes* and ever codon optimized for expression in the fungus *Aspergillus aculeatus* and *A. niger*. Recently, advanced technology of CRISPR/Cas with ribonucleoprotein complexes and transiently selected telomere vectors was reported to allow highly efficient marker-free and multiple genome editing in *Botrytis cinerea* (Leisen et al., 2020; Zou et al., 2021), which is also desirable for the edible fungus. Thus, there is still much to be learned and optimized for the use of CRISPR/Cas9 in *P. cocos.*

The sgRNA-CRISPR-Cas tool causes deleterious off-target effects at the genomic level. Up until now, dozens of tools and software have been developed to predict off-target mutations, along with improved on-target efficiency to ameliorate the off-target effects (Naeem et al., 2020). While most of the CRISPR-Cas9 off-target predictor is online for the model organism with precise genome information (Naeem et al., 2020). Unfortunately, most of the valuable species, just like *P. cocos*, is not a model organism. It is not convenient to harness CRISPR-Cas9 to edit target genes and devise efficient and specific sgRNA using these available tools for these non-model organisms, as well as the prediction of the off-target sites. To overcome this problem, we attempted to use the offline software Tbtools to predict the potential off-target sites in *P. cocos* with the availability of its genome information. By this way, an off-target was successfully detected, indicating that the development of method of using offline software enabling *in vitro* prediction of off-target is a promising alternative (Qin et al., 2017).

## Conclusion

This is the first report of a successful CRISPR system developed in the *P. cocos* with endogenous U6 promoter, which could greatly raise hope for the molecular breeding development of this traditional edible and medicinal fungus. Our study will also open the way for the development of various CRISPR-Cas-based applications integrating genome-wide off-target prediction and detection in non-model filamentous fungi.

## Data availability statement

The original contributions presented in this study are included in the article/Supplementary material, further inquiries can be directed to the corresponding author.

## Author contributions

ZX and CZ performed the experiments, analyzed the data, and wrote the manuscript. XL and ZW contributed to the experiments and data analysis. RZ and JX helped on the collection of the samples and data analysis. SZ contributed to the experiment design. JJ conceived the project and directed the research. All authors have read and agreed to the published version of the manuscript.

## Footnotes

1
https://bioinformatics.psb.ugent.be/webtools/plantcare/html/

2
https://portals.broadinstitute.org/gpp/public/

## References

[B1] Arazoe T. Ogawa T. Miyoshi K. Yamato T. Ohsato S. Sakuma T. (2015). Tailor-made CRISPR/Cas system for highly efficient targeted gene replacement in the rice blast fungus. *Biotechnol. Bioeng.* 112 1335–1342. 10.1002/bit.25662 25683503

[B2] Boonanuntanasarn S. Panyim S. Yoshizaki G. (2009). Characterization and organization of the U6 snRNA gene in zebrafish and usage of their promoters to express short hairpin RNA. *Mar. Genomicss* 1 115–121. 10.1016/j.margen.2008.10.001 21798162

[B3] Brandfass C. Karlovsky P. (2008). Upscaled CTAB-based DNA extraction and real-time PCR assays for Fusarium culmorum and F. graminearum DNA in plant material with reduced sampling error. *Int. J. Mol. Sci.* 9 2306–2321. 10.3390/ijms9112306 19330077 PMC2635625

[B4] Cao S. Yang Y. Bi G. Nelson D. Hu S. Makunga N. P. (2021). Genomic and transcriptomic insight of giant sclerotium formation of wood-decay fungi. *Front. Microbiol.* 12:746121. 10.3389/fmicb.2021.746121 34712214 PMC8546338

[B5] Chen B. X. Wei T. Ye Z. W. Yun F. Kang L. Z. Tang H. B. (2018). Efficient CRISPR-Cas9 gene disruption system in edible-medicinal mushroom Cordyceps militaris. *Front. Microbiol.* 9:1157. 10.3389/fmicb.2018.01157 29946301 PMC6005869

[B6] Chen C. Chen H. Zhang Y. Thomas H. R. Frank M. H. He Y. (2020). TBtools: An integrative toolkit developed for interactive analyses of big biological data. *Mol. Plant* 13 1194–1202. 10.1016/j.molp.2020.06.009 32585190

[B7] Denes C. E. Cole A. J. Cole A. J. Li G. Neely G. G. Hesselson D. (2021). Approaches to enhance precise CRISPR/Cas9-mediated genome editing. *Int. J. Mol. Sci.* 22:8571. 34445274 10.3390/ijms22168571 PMC8395304

[B8] Deng H. Gao R. Liao X. Cai Y. (2017). Genome editing in *Shiraia bambusicola* using CRISPR-Cas9 system. *J. Biotechnol.* 259 228–234. 10.1016/j.jbiotec.2017.06.1204 28690135

[B9] Floudas D. Binder M. Riley R. Barry K. Blanchette R. A. Henrissat B. (2012). The Paleozoic origin of enzymatic lignin decomposition reconstructed from 31 fungal genomes. *Science* 336:1715. 10.1126/science.1221748 22745431

[B10] Gao Z. Herrera-Carrillo E. Berkhout B. (2018). RNA polymerase II activity of type 3 Pol III promoters. *Mol. Ther. Nucl. Acids* 12 135–145. 10.1016/j.omtn.2018.05.001 PMC6023835 30195753

[B11] He H. Guo J.-Y. Shu S.-H. Wang M. (2016). Cloning and bioinformatics analysis of cytochrome P450 reductase gene in *Poria cocos*. *Chin. Tradit. Herbal Drugs* 47 2909–2915.

[B12] Hu S. Ni W. Hazi W. Zhang H. Zhang N. Meng R. (2011). Cloning and functional analysis of sheep U6 promoters. *Anim. Biotechnol.* 22 170–174. 10.1080/10495398.2011.580669 21774625

[B13] Jia X. Ma L. Li P. Chen M. He C. (2016). Prospects of *Poria cocos* polysaccharides: Isolation process, structural features and bioactivities. *Trends Food Sci. Technol.* 54 52–62.

[B14] Jin J. Liu H. Zhong C. Cheng M. Xie J. Xie Z. (2020a). Historical view of biological recognition, production and evaluation of medicinal fungus *Poria cocos*. *Mod. Chin. Med.* 22 1888–1895.

[B15] Jin J. Zhong C. Xie J. Liu H. Liang X. Ye H. (2020b). Processing technology and products of *Poria cocos* in China. *Mod. Chin. Med.* 22 1441–1446.

[B16] Jin J. Zhong C. Chi X. Xie J. Deng J. Dai J. (2022). Insights into international trade and quality standards of *Poria cocos* in main target countries and regions. *Mod. Chin. Med.* 24 344–351.

[B17] Kim J.-H. Sim H. A. Jung D. Y. Lim E. Y. Kim Y. T. Kim B. J. (2019). *Poria cocos* Wolf extract ameliorates hepatic steatosis through regulation of lipid metabolism, inhibition of ER stress, and activation of autophagy via AMPK activation. *Int. J. Mol. Sci.* 20:4801. 10.3390/ijms20194801 31569635 PMC6801774

[B18] Lambeth L. S. Moore R. J. Muralitharan M. Dalrymple B. P. Mcwilliam S. Doran T. J. (2005). Characterisation and application of a bovine U6 promoter for expression of short hairpin RNAs. *BMC Biotechnol.* 5:13. 10.1186/1472-6750-5-13 15885150 PMC1142307

[B19] Le C. Ran F. A. Cox D. Lin S. Barretto R. (2013). Multiplex genome engineering using CRISPR/Cas systems. *Science* 339:1231143. 10.1126/science.1231143 PMC3795411 23287718

[B20] Lee S. Choi E. Yang S.-M. Ryoo R. Moon E. Kim S.-H. (2018). Bioactive compounds from sclerotia extract of *Poria cocos* that control adipocyte and osteoblast differentiation. *Bioorg. Chem.* 81 27–34. 10.1016/j.bioorg.2018.07.031 30092384

[B21] Leisen T. Bietz F. Werner J. Wegner A. Hahn M. (2020). CRISPR/Cas with ribonucleoprotein complexes and transiently selected telomere vectors allows highly efficient marker-free and multiple genome editing in Botrytis cinerea. *PLoS Pathog.* 16:e1008326. 10.1371/journal.ppat.1008326 32804988 PMC7451986

[B22] Lescot M. Déhais P. Thijs G. Marchal K. Moreau Y. Peer Y. V. D. (2002). PlantCARE, a database of plant cis-acting regulatory elements and a portal to tools for in silico analysis of promoter sequences. *Nucleic Acids Res.* 30 325–327. 10.1093/nar/30.1.325 11752327 PMC99092

[B23] Li C. Brant E. Budak H. Zhang B. (2021a). CRISPR/Cas: A Nobel Prize award-winning precise genome editing technology for gene therapy and crop improvement. *J. Zhejiang Univ. Sci. B* 22 253–284. 10.1631/jzus.B2100009 33835761 PMC8042526

[B24] Li X. Zuo X. Li M. Yang X. Zhi J. Sun H. (2021b). Efficient CRISPR/Cas9-mediated genome editing in *Rehmannia glutinosa*. *Plant Cell Rep.* 40 1695–1707. 10.1007/s00299-021-02723-3 34086068

[B25] Li Q. Wang W. Zhao N. Li P. Xin Y. Hu W. (2017). Identification and validation of a *Schistosoma japonicum* U6 promoter. *Parasite. Vector.* 10:281. 10.1186/s13071-017-2207-4 28583151 PMC5460494

[B26] Ling K. Chen H. Zhang W. He S. Xiong Z. Zhang Y. (2017). Building a genetic manipulation tool box for orchid biology: Identification of constitutive promoters and application of CRISPR/Cas9 in the orchid. *Dendrobium officinale*. *Front. Plant Sci.* 7:2036. 10.3389/fpls.2016.02036 28127299 PMC5226938

[B27] Long L. Guo D. D. Gao W. Yang W. W. Hou L. P. Ma X. N. (2018). Optimization of CRISPR/Cas9 genome editing in cotton by improved sgRNA expression. *Plant Methods* 14:85. 10.1186/s13007-018-0353-0 30305839 PMC6169012

[B28] Luo H. Qian J. Xu Z. Liu W. Xu L. Li Y. (2020). The *Wolfiporia cocos* genome and transcriptome shed light on the formation of its edible and medicinal sclerotium. *Genomi. Proteom. Bioinf.* 18 455–467. 10.1016/j.gpb.2019.01.007 33359677 PMC8242266

[B29] Ma X. Chen L. Zhu Q. Chen Y. Liu Y.-G. (2015). Rapid decoding of sequence-specific nuclease-induced heterozygous and biallelic mutations by direct sequencing of PCR products. *Mol. Plant* 8 1285–1287. 10.1016/j.molp.2015.02.012 25747846

[B30] Naeem M. Majeed S. Hoque M. Z. Ahmad I. J. C. (2020). Latest developed strategies to minimize the off-target effects in CRISPR-Cas-mediated genome editing. *Cells* 9:1608. 10.3390/cells9071608 32630835 PMC7407193

[B31] Nawrocki E. P. Kolbe D. L. Eddy S. R. (2009). Infernal 1.0: Inference of RNA alignments. *Bioinformatics* 25 1335–1337. 10.1093/bioinformatics/btp157 19307242 PMC2732312

[B32] Nidhi S. Anand U. Oleksak P. Tripathi P. Lal J. A. Thomas G. (2021). Novel CRISPR–Cas systems: An updated review of the current achievements, applications, and future research perspectives. *Int. J. Mol. Sci.* 22:3327. 10.3390/ijms22073327 33805113 PMC8036902

[B33] Nie A. Chao Y. Zhang X. Jia W. Zhou Z. Zhu C. (2020). Phytochemistry and pharmacological activities of *Wolfiporia cocos* (F.A. Wolf) Ryvarden and Gilb. *Front. Pharmacol.* 11:505249. 10.3389/fphar.2020.505249 33071776 PMC7533546

[B34] Nødvig C. S. Nielsen J. B. Kogle M. E. Mortensen U. H. (2015). A CRISPR-Cas9 system for genetic engineering of filamentous fungi. *PLoS One* 10:e0133085. 10.1371/journal.pone.0133085 26177455 PMC4503723

[B35] Pavese V. Moglia A. Abbà S. Milani A. M. Marinoni D. T. Corredoira E. (2022). First report on genome editing via ribonucleoprotein (RNP) in Castanea sativa Mill. *Int. J. Mol. Sci.* 23:5762. 10.3390/ijms23105762 35628572 PMC9145500

[B36] Qin H. Xiao H. Zou G. Zhou Z. Zhong J.-J. (2017). CRISPR-Cas9 assisted gene disruption in the higher fungus *Ganoderma* species. *Process Biochem.* 56 57–61.

[B37] Schuster M. Schweizer G. Reissmann S. Kahmann R. (2016). Genome editing in *Ustilago maydis* using the CRISPR–Cas system. *Fungal Genet. Biol.* 89 3–9. 26365384 10.1016/j.fgb.2015.09.001

[B38] Singh D. Sternberg S. H. Fei J. Doudna J. A. Ha T. (2016). Real-time observation of DNA recognition and rejection by the RNA-guided endonuclease Cas9. *Nat. Commun.* 7:12778. 10.1038/ncomms12778 27624851 PMC5027287

[B39] Song R. Zhai Q. Sun L. Huang E. Zhang Y. Zhu Y. (2019). CRISPR/Cas9 genome editing technology in filamentous fungi: Progress and perspective. *Appl. Microbiol. Biot.* 103 6919–6932. 10.1007/s00253-019-10007-w PMC6690858 31332488

[B40] Sun Q. Wei W. Zhao J. Song J. Peng F. Zhang S. (2015). An efficient PEG/CaCl2-mediated transformation approach for the medicinal fungus *Wolfiporia cocos*. *J. Microbiol. Biotechn.* 25 1528–1531. 10.4014/jmb.1501.01053 26017228

[B41] Wang C. Rollins J. A. (2021). Efficient genome editing using endogenous U6 snRNA promoter-driven CRISPR/Cas9 sgRNA in Sclerotinia sclerotiorum. *Fungal Genet. Biol.* 154:103598. 10.1016/j.fgb.2021.103598 34119663

[B42] Wang J. R. Lin J. F. Guo L. Q. You L. F. Zeng X. L. Wen J. M. (2014b). Cloning and characterization of squalene synthase gene from *Poria cocos* and its up-regulation by methyl jasmonate. *J. Microbiol. Biotechn.* 30:613. 10.1007/s11274-013-1477-z 24030169

[B43] Wang J. Li Y. Liu D. (2014a). Cloning and characterization of farnesyl diphosphate synthase gene involved in triterpenoids biosynthesis from *Poria cocos*. *Int. J. Mol. Sci.* 15 22188–22202. 10.3390/ijms151222188 25474088 PMC4284702

[B44] Wang P. A. Xiao H. Zhong J. J. (2020). CRISPR-Cas9 assisted functional gene editing in the mushroom *Ganoderma lucidum*. *Appl. Microbiol. Biot.* 104 1661–1671. 10.1007/s00253-019-10298-z 31865439

[B45] Wang Y. Z. Zhang J. Zhao Y. L. Li T. Shen T. Li J. Q. (2013). Mycology, cultivation, traditional uses, phytochemistry and pharmacology of *Wolfiporia cocos* (Schwein.) Ryvarden et Gilb.: A review. *J. Ethnopharmacol.* 147 265–276. 10.1016/j.jep.2013.03.027 23528366

[B46] Xing H.-L. Dong L. Wang Z.-P. Zhang H.-Y. Han C.-Y. Liu B. (2014). A CRISPR/Cas9 toolkit for multiplex genome editing in plants. *BMC Plant Biol.* 14:327. 10.1186/s12870-014-0327-y 25432517 PMC4262988

[B47] Xuan L. Wu S. Jiao X. Sui C. Wei J. (2017). Application of CRISPR/Cas9 in plant biology. *Acta Pharm. Sin. B* 7 292–302. 28589077 10.1016/j.apsb.2017.01.002 PMC5443236

[B48] Zhang N. He J. Muhammad A. Shao Y. (2022). CRISPR/Cas9–mediated genome editing for *Pseudomonas* fulva, a novel *Pseudomonas* species with clinical, animal, and plant–associated isolates. *Int. J. Mol. Sci.* 23:5443. 10.3390/ijms23105443 35628253 PMC9145825

[B49] Zhang X. Xu Z. C. Xu J. Ji A. J. Luo H. M. Song J. Y. (2016). Selection and validation of reference genes for normalization of quantitative real-time reverse transcription PCR analysis in *Poria cocos* (Schw.) Wolf (Fuling). *Chin. Med.* 11:8. 10.1186/s13020-016-0079-8 26937250 PMC4774131

[B50] Zhou Z. Tan H. Li Q. Chen J. Gao S. Wang Y. (2018). CRISPR/Cas9-mediated efficient targeted mutagenesis of RAS in *Salvia miltiorrhiza*. *Phytochemistry* 148 63–70. 10.1016/j.phytochem.2018.01.015 29421512

[B51] Zou G. Xiao M. Chai S. Zhu Z. Wang Y. Zhou Z. (2021). Efficient genome editing in filamentous fungi via an improved CRISPR-Cas9 ribonucleoprotein method facilitated by chemical reagents. *Microb. Biotechnol.* 14 2343–2355. 10.1111/1751-7915.13652 32841542 PMC8601184

