# Supplementary material for: Genome editing in the edible fungus Poria cocos using CRISPR-Cas9 system integrating genome-wide off-target prediction and detection
Source: Front Microbiol. 2022 Aug 22;13:966231. doi: 10.3389/fmicb.2022.966231 (PMC9441760; doi:10.3389/fmicb.2022.966231)
Supplement: Supplementary file 1 [file Data_Sheet_1.docx]

Supplementary Material

**Supplementary Figure 1.** Comparison of the U6 snRNA genes of *Poria cocos* and *Aspergillus nidulans* by alignment.
